# Freshwater transfer affected intestinal microbiota with correlation to cytokine gene expression in Asian sea bass

**DOI:** 10.3389/fmicb.2023.1097954

**Published:** 2023-04-06

**Authors:** Syed Monzur Morshed, Yu-Yi Chen, Chia-Hao Lin, Yen-Po Chen, Tsung-Han Lee

**Affiliations:** ^1^Department of Life Sciences, National Chung Hsing University, Taichung, Taiwan; ^2^The iEGG and Animal Biotechnology Center, National Chung Hsing University, Taichung, Taiwan; ^3^Department of Animal Science, National Chung Hsing University, Taichung, Taiwan; ^4^Department of Marine Biotechnology, National Kaohsiung University of Science and Technology, Kaohsiung, Taiwan

**Keywords:** Asian sea bass, gut microbiota, mucosa, digesta, cytokine, salinity

## Abstract

As a catadromous fish, Asian sea bass (*Lates calcarifer*) juveniles migrate from seawater (SW) to freshwater (FW) for growth and development. During migration, they undergo physiological changes to acclimate to environmental salinity. Thus, it is crucial to understand how SW-to-FW migration affects the gut microbiota of catadromous fish. To the best of our knowledge, no study has revealed the effects of transfer to hypotonic environments on a catadromous fish microbiota. In this study, we aimed to determine the effects of FW transfer on the microbiota and cytokine gene expression in the intestines of juvenile catadromous Asian sea bass. The relationship between the water and the gut microbiota of this euryhaline species was also examined. We found that FW transfer affected both mucosa- and digesta-associated microbiota of Asian sea bass. *Plesiomonas* and *Cetobacterium* were dominant in both the mucosa- and digesta-associated microbiota of FW-acclimated sea bass. The pathogenic genera *Vibrio*, *Staphylococcu*s, and *Acinetobacter* were dominant in the SW group. Although dominant fish microbes were present in the water, fish had their own unique microbes. Vitamin B6 metabolism was highly expressed in the FW fish microbiota, whereas arginine, proline, and lipid metabolism were highly expressed in the SW fish microbiota. Additionally, the correlation between cytokine gene expression and microbiota was found to be affected by FW transfer. Taken together, our results demonstrated that FW transfer altered the composition and functions of mucosa- and digesta-associated microbiota of catadromous Asian sea bass intestines, which correlated with cytokine gene expression.

## 1. Introduction

Fish gut microbiota is important for maintaining physiological homeostasis and disease resistance against pathogens (Wang et al., 2018). Gut microbiota includes bacteria, viruses, and fungi in the intestinal environment. However, in fish, studies to date have focused mainly on bacteria (Wang et al., 2018). As water inhabitants, fish are always in close contact with changing environmental stimuli. Environmental abiotic factors, such as temperature, salinity, and pH, affect the fish gut microbiota and immune response (Sylvain et al., 2016; Dehler et al., 2017; Behera et al., 2022; Steiner et al., 2022). Recently, advanced sequencing technology and developed bioinformatics tools have increased the research on the fish gut microbiota (Ghanbari et al., 2015). More studies are needed now to elucidate the mechanisms involved in host-microbiome interaction in fish (López Nadal et al., 2020).

Salinity is an abiotic factor reported to affect the fish microbiota (Wong and Rawls, 2012; Tarnecki et al., 2017). However, the relationship between salinity adaptation and microbiome modulation remains unclear. Some euryhaline fish move from environments of one salinity level to another during their lifetime for growth and maturation (Pelletier et al., 2009). Hence, it is crucial to understand the mechanisms of acclimation and microbial dynamics in euryhaline fish under extreme salinities. Many studies have focused on osmoregulatory mechanisms and energy metabolism of euryhaline fish at different salinities (Hwang and Lee, 2007; Hu et al., 2015), but very few studies have emphasized the microbial interactions and physiological changes that occur during salinity acclimation. SW transfer has been reported to alter the intestinal and skin microbiota of anadromous Atlantic salmon (*Salmo salar*) (Lokesh and Kiron, 2016; Dehler et al., 2017; Rudi et al., 2018; Jaramillo-Torres et al., 2019; Wang et al., 2021). In addition, it was found that hypotonic stress induced gill and gut microbiota changes in marine medaka (*Oryzias melastigma*) and the authors hypothesized that changes in the microbiota may play a role in salinity acclimation (Lai et al., 2020, 2022). Hypoosmotic stress has also been found to reduce bacterial diversity and increase pathogenic bacteria in the yellowfin seabream (*Acanthopagrus latus*) (Lin et al., 2020). On the other hand, salinity stress increases opportunistic bacteria and decreases beneficial bacteria in the Nile tilapia (*Oreochromis niloticus*) (Zhang et al., 2016). In contrast, (Zheng et al. (2019) reported a similar composition of gut microbiota in FW- and SW-acclimated Asian sea bass, which is contrary to the existing literature. Two types of microbiota have been reported in the fish gut: autochthonous and allochthonous. Autochthonous microbes remain in close contact with the tissue and withstand bile acids, whereas allochthonous microbes remain in stool in a transient state (Nayak, 2010). It is assumed that autochthonous microbes found in the gut mucosa are more important for the host-microbe interactions because they colonize the intestine (Egerton et al., 2018). Therefore, it is important to separate the mucosa- and digesta-associated microbiota under salinity stress. To the best of our knowledge, the effects of salinity on the mucosa- and digesta-associated microbiota of euryhaline teleost have not been studied independently.

The role of water microbiota on fish gut microbiota change has also been an important research question. In Atlantic salmon hatcheries, the built-in water environment microbiota was found to affect the fish microbiota (Minich et al., 2020). It was also reported that the physiochemical parameters of the water affected the microbiota of tilapia larvae with a correlation to water microbiota (Giatsis et al., 2015). In addition, when the gut microbiota of silver carp (*Hypophthalmichthys molitrix*), grass carp (*Ctenopharyngodon idella*), bighead carp (*Hypophthalmichthys nobilis*), and goldfish (*Carassius auratus*) were compared to the water microbiota of their natural habitats, a strong correlation was found between gut and water microbiota (Zeng et al., 2020). In contrast, a study on Mexican mollies (*Poecillia mexicana*) revealed that water microbiota did not correlate with fish microbiota because the major operational taxonomic unit (OTU) in fish microbiota could not be found in the water (Schmidt et al., 2015). To date, most studies have suggested that the water microbiota affects fish microbiota, although some studies have suggested the opposite.

Salinity was found to affect the immune responses of Asian sea bass including immune gene expression, lysozyme activity, ACH50 activity, and total Ig abundance (Delamare-Deboutteville et al., 2006; Xia et al., 2013; Mozanzadeh et al., 2020). It was reported that IgM, peroxidase content, and alternative complement activity varied with salinity in gilthead seabream (*Sparus auratus*) and the authors hypothesized that osmoregulatory hormones, such as prolactin, growth hormone, and cortisol, might play roles in salinity-dependent immune responses (Cuesta et al., 2005). Analyses of the gill transcriptome of SW- and FW-acclimated Japanese eels (*Anguilla japonica*) revealed differentially regulated immune genes. Among them, C-reactive protein, toll-like receptor 2, and IL-1R2 levels were significantly higher in FW-acclimated eels than in SW-acclimated eels (Gu et al., 2018).

Asian sea bass is a catadromous fish. Adult fish migrate from FW to SW for maturity and spawning, and larvae and juveniles return from SW to FW for development and growth. Therefore, salinity is a crucial factor in the maturation, reproduction, and spawning of the Asian sea bass (Jerry, 2013). Asian sea bass is now an important aquaculture species that can be cultured in the water of different salinities owing to their euryhalinity (Jerry, 2013). Studying the mechanisms underlying the effects of salinity on the physiology and gut microbiota of this species and the correlation between immune response and microbiota would provide a basis for aquaculture practice. As a catadromous fish, it is important to know how SW-to-FW transfer affects the gut microbiota of Asian sea bass, with a specific focus on mucosa- and digesta-associated microbiota because they may play differential roles in host physiology. This study examined the effects of FW transfer on the mucosa- and digesta-associated microbiota of the Asian sea bass. In addition, the water microbiota was analyzed to determine whether it was correlated with the gut microbiota after FW transfer. This study also aimed to compare the mRNA expression of pro- and anti-inflammatory cytokines and their correlations with microbiota abundance in Asian sea bass guts between different salinity groups. To the best of our knowledge, this is the first study to examine the effect of FW transfer on the gut microbiota of a catadromous fish with an emphasis on both mucosa- and digesta-associated microbiota. The findings of this study will also shed light on the interaction between hosts and microbes in aquatic environments by analyzing the correlation between the expression of immune genes and microbiota in the guts of Asian sea bass after FW transfer.

## 2. Materials and methods

### 2.1. Experimental animal and environments

Juvenile Asian sea bass was purchased from a fish farm in Changhua County, Taiwan. The average final full length and body weight of total fish was 19.81 ± 0.39 cm and 110.33 ± 6.43 g, respectively. All the experimental fish were reared in tanks with a recirculatory system. Each day, the fish were fed once to satiation with commercial pellets. The water temperature was maintained at 28 ± 1°C. A 12-h dark-light photoperiod was maintained throughout the experiment (the light period was from 8:00 to 20:00). A total of 13 fish were first reared in SW (35 ‰) for one month, and six of them were subsequently transferred to the FW tank directly. FW-transferred fish were sampled after one month. SW fish were also sampled simultaneously. SW was prepared using tap water mixed with Blue Treasure Tropic Fish Sea salt (Qingdao, China). The experimental protocol was reviewed and approved by the Institutional Animal Care and Use Committee (IACUC No. 108-137) of the National Chung Hsing University, Taichung, Taiwan.

### 2.2. Experimental design

In the present study, we compared the effects of FW transfer on microbiota and cytokine gene expression in the intestines of Asian sea bass. Six individuals from both SW and FW environments were used for mucosa- and digesta-associated microbiota analyses. The same six individuals were also used for analyzing expression of cytokine genes. FW and SW environmental samples were collected for water microbiota analysis. Intestinal samples from the Asian sea bass were separated into two parts: the first segment consisted of the anterior section, and the second segment consisted of both the middle and posterior sections. Previous research has found that enzyme-producing bacteria are the most abundant in the latter parts of the intestine; therefore, the second segment of the intestine was used for microbiota analysis (Shcherbina and Kazlawlene, 1971; Das et al., 2014). The mucosa (intestinal tissues) and digesta (intestinal contents) of intestinal samples were collected separately. The first segments of the intestines were also collected from the same individuals to determine cytokine gene expression in the same group of fish.

### 2.3. Sample preparation

The juvenile Asian sea bass was first anesthetized with 0.04% (400 μl/L) 2-phenoxyethanol (PANREAC, Barcelona, Spain) and then sacrificed immediately for sampling. For gut microbiota analysis, the second segment (containing the middle and posterior sections) of the intestine was sampled. The samples were then divided into two parts: digesta (gut content) and mucosa (gut tissue). The digesta sample was collected by carefully squeezing with sterilized tweezers, and mucosa samples were further cleaned to confirm that no digesta was left in the intestine. All sampling processes were performed in a laminar flow hood using an aseptic technique. Digesta and mucosa samples were kept separately in a 2 ml sterilized microcentrifuge tube and frozen at −80°C until further analysis. For water microbiota analysis, 500 ml of water was vacuum-filtered with 0.22 μm filter paper (Pall Corporation, Port Washington, NY, USA). The filter was then cut into small pieces with sterilized scissors and placed into a 2 ml sterilized microcentrifuge tube. DNA extraction was performed on the filter samples for subsequent analysis of the water microbiota.

For qPCR analysis, the first segment of intestinal tissue was sampled, maintained in RNAlater, and snapped frozen in liquid nitrogen. The tissues were then stored at −80°C before analysis.

### 2.4. Microbiota DNA extraction and PCR

Total genomic DNA was extracted using a QIAamp^®^ PowerFecal^®^ DNA kit (QIAGEN, Hilden, Germany) according to the manufacturer’s guidelines. DNA concentration was measured using an Equalbit dsDNA HS Assay Kit (Vazyme, Nanjing, China).

### 2.5. Amplicon generation, library preparation, and sequencing

Approximately 20–30 ng of DNA was used to generate amplicons. The V3 and V4 hypervariable regions of the 16S RNA were selected for amplification. The V3 and V4 regions were amplified using the forward primer “CCTACGGRRBGCASCAGKVRVGAAT” and reverse primer “GGACTACNVGGGTWTCTAATCC” (Zheng et al., 2019). Simultaneously, indexed adapters were added to the ends of the 16S rRNA amplicons to generate indexed libraries for downstream NGS sequencing. PCR reaction was performed in triplicate with 25 μl mixture containing 2.5 μl of TransStart^®^ Buffer (Biotrend, Köln, Germany), 2 μl of dNTPs, 1 μl of each primer, and 20 ng of template DNA.

DNA library concentration was validated using a Qubit 3.0 Fluorometer (Thermo Fisher Scientific, Wilmington, DE, USA). The library was quantified to 10 nM, and DNA libraries were multiplexed and loaded on an Illumina MiSeq instrument, according to the manufacturer’s instructions (Illumina, San Diego, CA, USA). Finally, paired-end sequencing was performed, and image analysis and base calling were conducted using the control software embedded in the instrument.

### 2.6. Microbiota data analyses

Sequencing analyses were conducted by GENEWIZ, Inc. (South Plainfield, NJ, USA) using the following procedure. First, adapters, low-quality data, chimeric sequences, and singletons were removed from the raw sequences. Mitochondrial and chloroplast sequences were also removed. Sequences were grouped with 97% similarity using vsearch (1.9.6). Data were analyzed using QIIME against the Silva database (version 138) (Caporaso et al., 2010; Quast et al., 2012). Good’s coverage for all the samples was greater than 0.99. Alpha diversity was calculated from the rarified data using the QIIME. Beta diversity was analyzed using QIIME by creating a phylogenetic tree. PCoA plot and ANOSIM analyses were performed using the R software (McMurdie and Holmes, 2013). Predictive functional analysis was done by PICRUSt (Langille et al., 2013).

### 2.7. Total RNA extraction and reverse transcription

Total RNA from intestinal tissue was extracted using TRI reagent^®^ (Molecular Research Center, Inc., Cincinnati, OH, USA) according to the manufacturer’s instructions. The RNA pellet was dissolved in 20 μl DEPC water. RNA integrity and concentration were checked using a NanoDrop 2000 (Thermo Fisher Scientific, Wilmington, DE, USA). The A260/A230 ratio was over 2.0, and the A260/A280 ratio was between 1.9 and 2.0 for all RNA samples. RNA integrity was further checked by 0.6% agarose gel (SeaKem^®^ LE Agarose; Lonza, Basel, Switzerland) electrophoresis with the Safeview™ classic (ABM, San Jose, CA, USA) nucleic acid stain. Reverse transcription was performed using the iScript™ cDNA synthesis kit (Bio-Rad, Hercules, CA, USA) according to the manufacturer’s protocol using 1 μg of total RNA.

### 2.8. Real-time PCR

The mRNA levels of target genes were quantified using Rotor-Gene Q Real-Time PCR (QIAGEN, Hilden, Germany). *Elongation factor 1α* (*elf1α*) was used as the housekeeping gene to normalize gene expression because it is one of the most stable reference genes for Asian sea bass under salinity change, fasting challenge, and bacterial infection (De Santis et al., 2011; Paria et al., 2016; Azodi et al., 2021). One intestinal sample of FW Asian sea bass was used as an internal control. In the qPCR reaction mixture, 10 μL Kapa SYBR^®^ Fast qPCR master mix (2×) kit (Merck, Cape Town, South Africa), 200–300 nM primer, 1 μl diluted cDNA, and autoclaved double distilled water were added up to 20 μL in volume. Primers were designed using primer3plus^1^ in the conserved region and compared to other teleost sequences. Next, primers were checked for their specific products using Primer BLAST software.^2^ Primer specificity was further checked by running a melting curve, a single PCR product on 2% agarose gel, and at last sequencing the PCR product. Primer efficiencies ranged from 95 to 105% (Supplementary Table 1). The relative expression of target genes was calculated using the relative C_t_ method (Livak and Schmittgen, 2001) using the following formula: relative expression = 2^∧^−[(Ct_t *target gene*, n–_Ct _*elf*1α, n_)_–_(Ct_t *target gene*, c_−Ct _*elf*1α, c_)]. In the formula, “Ct” corresponds to the threshold cycle number, “n” indicates each cDNA sample used in these experiments and “c” indicates the control.

### 2.9. Statistical analyses

Alpha diversity metrics, Chao 1, and Shannon indices of fish microbiota were analyzed using Kruskal–Wallis with Dunn’s multiple comparisons test. Correlation analysis between microbiota and cytokine gene expression was performed using Spearman’s correlation analysis. All statistical analyses were performed using the GraphPad Prism software (version 9.2.0). All values were expressed as means ± standard error of the mean (SEM), and statistical significance was set at *P* < 0.05.

## 3. Results

### 3.1. Alpha and beta diversity

**FIGURE 1:**
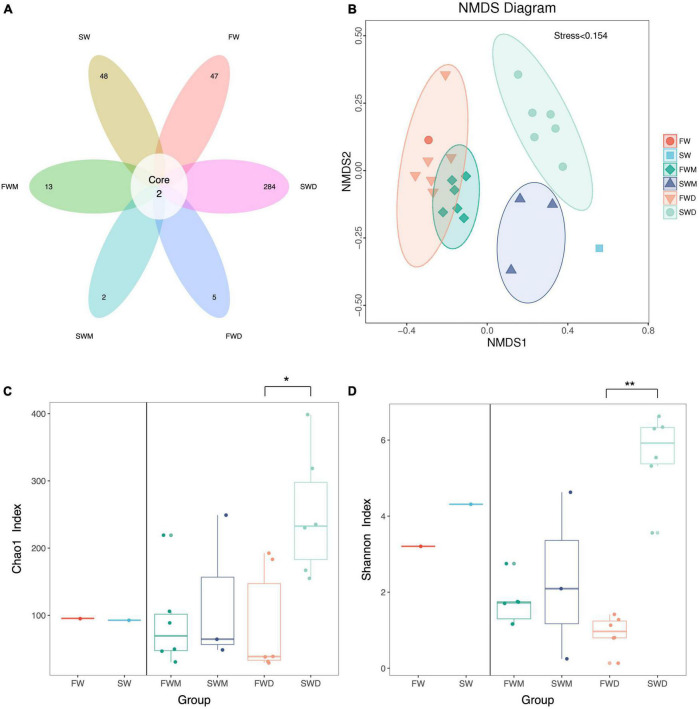
Alpha and beta diversity of gut microbiota of Asian sea bass. **(A)** The Venn diagram revealed core and unique operation taxonomic units (OTUs) among different groups. **(B)** The NMDS plot revealed beta diversity between different groups. **(C)** Chao 1 and **(D)** Shannon index revealed alpha diversity. SW, seawater; FW, freshwater; FWM, freshwater mucosa samples; SWM, seawater mucosa samples; FWD, freshwater digesta samples; SWD, seawater digesta samples.

A total of 875,376 paired-end read quality sequences were obtained after removing the chimeric sequences. Following microbiota data analysis, 814 OTU were found with a 97% similarity level. Only two OTU were shared between the groups: OTU 1 and OTU 96 (Figure 1A and Supplementary Table 2). Environmental microbiota samples (FW and SW) and fish microbiota samples, freshwater mucosa (FWM), seawater mucosa (SWM), freshwater digesta (FWD), and seawater digesta (SWD) had 47, 48, 13, 2, 5, and 284 unique OTUs, respectively (Figure 1A). Good’s coverage for all samples reached close to 1. The flattening of the rarefaction curve indicated that sequencing was deep enough to cover all OTUs present in the samples (Supplementary Figure 1). The NMDS plot revealed that samples from the same group were clustered together, indicating that salinity affected the gut microbiota of fish. The mucosa- and digesta-associated microbiota from the same salinity showed differences in clustering (Figure 1B). The Chao 1 index, indicating the richness of bacteria in a community, was significantly higher in SWD than in FWD (Figure 1C). The Shannon index, indicating the bacterial diversity in a community, was also significantly higher in SWD than in FWD (Figure 1D). Therefore, SWD was significantly high in terms of bacterial richness and diversity. The other groups did not differ significantly in terms of bacterial richness and diversity.

### 3.2. Salinity affected bacterial composition in the gut of Asian sea bass

**FIGURE 2:**
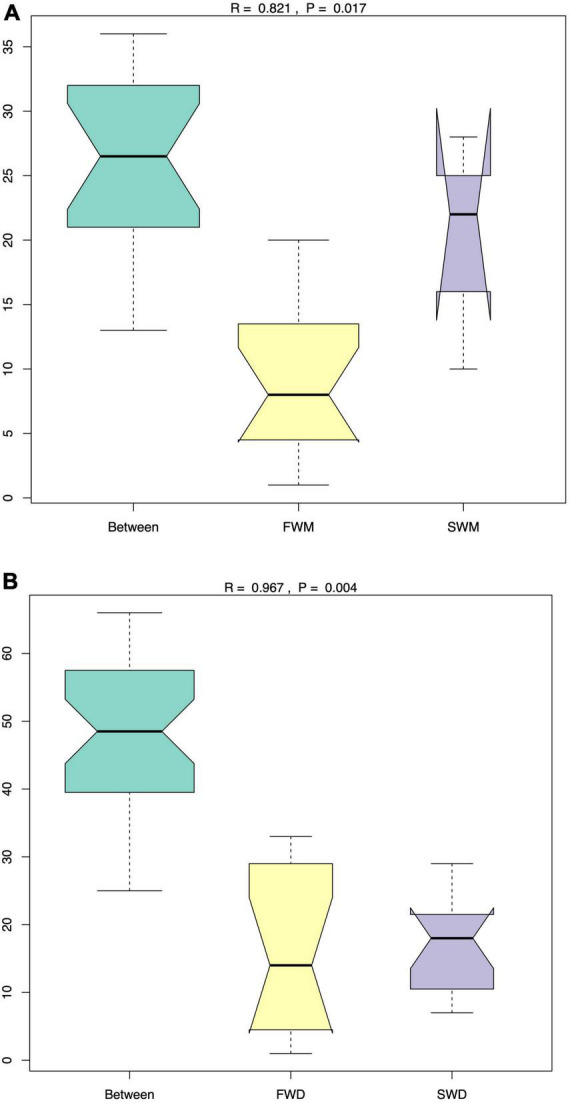
The ANOSIM analysis revealed that the **(A)** mucosa-associated microbiota and **(B)** digesta-associated microbiota were significantly different between FW and SW Asian sea bass. FWD, freshwater digesta samples; SWD, seawater digesta samples; FWM, freshwater mucosa samples; SWM, seawater mucosa samples.

**FIGURE 3:**
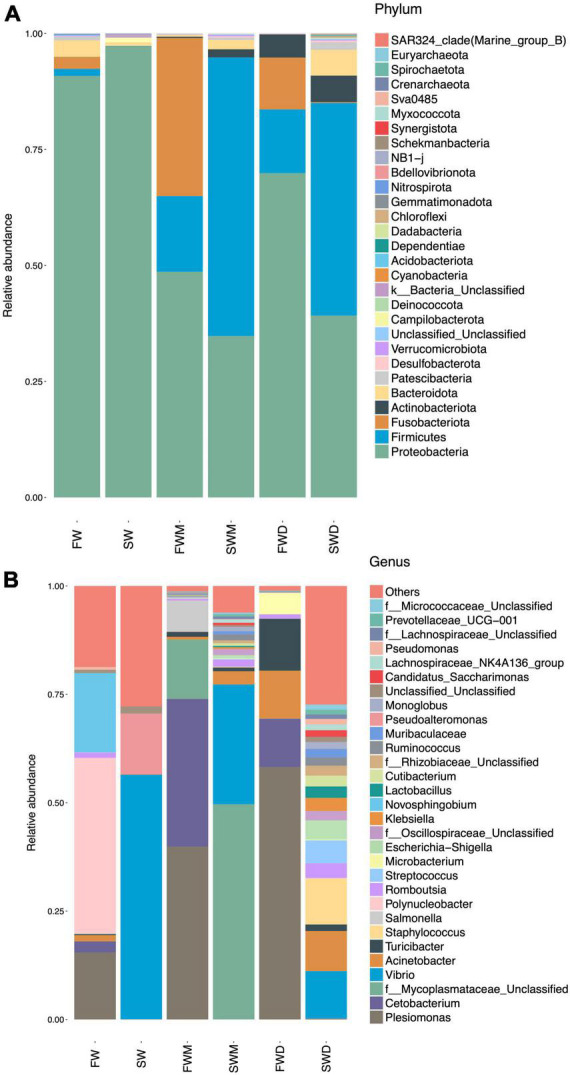
Relative abundance of major taxonomic lineages at the **(A)** phylum and **(B)** genus levels. SW, seawater; FW, freshwater; FWM, freshwater mucosa samples; SWM, seawater mucosa samples; FWD, freshwater digesta samples; SWD, seawater digesta samples.

ANOSIM analysis confirmed that FWM and SWM were significantly different, as the R values for both groups were close to 1. Similarly, the FWD and SWD were also significantly different (Figure 2). We analyzed the microbiota of both fish guts and environmental water. In the FW environment, Proteobacteria (90.85%), Bacteroidetes (3.54%), Fusobacteria (2.54%), and Firmicutes (1.54%) were dominant, whereas in the SW environment, Proteobacteria (97.27%) and Campilobacterota (1.04%) were dominant (Figure 3A). Proteobacteria was the most abundant phylum in the water microbiota.

Salinity altered both mucosa- and digesta-associated microbiota in the intestines of the Asian sea bass. At the phylum level, Proteobacteria (48.64%), Fusobacteria (34.06%), and Firmicutes (16.33%) were dominant in the mucosa-associated microbiota of FW fish, whereas Firmicutes (60.05%), Proteobacteria (34.79%), Bacteroidota (2.00%), and Actinobacteriota (1.75%) were dominant in the SW fish. The digesta of FW individuals were dominated by Proteobacteria (69.89%), Firmicutes (13.75%), Fusobacteria (11.17%), and Actinobacteriota (4.99%), whereas the digesta of SW individuals were dominated by Firmicutes (48.85%), Proteobacteria (39.19%), Actinobacteriota (5.71%), and Bacteroidota (5.54%) (Figure 3A and Supplementary Table 2). Thus, it was confirmed that salinity affected the dominant phyla and their abundances in the FW and SW groups. Moreover, mucosa- and digesta-associated microbiota differed in their dominant phyla and abundance even at the same salinity level.

At the genus level (Figure 3B and Supplementary Table 3), the FW environment was dominated by *Polynucleobacter* (40.52%), *Novoshingobium* (18.32%), and *Plesiomonas* (15.47%). The SW environment was dominated by *Vibrio* (56.36%) and *Pseudoalteromonas* (14.08%). Among the fish gut microbiota, the mucosa of FW fish was dominated by *Plesiomonas* (39.89%) and *Cetobacterium* (34.06%). The mucosa of SW fish was dominated by a genus from the families Mycoplasmataceae (49.65%) and *Vibrio* (27.62%). The digesta of FW fish was dominated by *Plesiomonas* (58.21%), *Cetobacterium* (34.69%), and *Turicibacter* (11.50%). The digesta of SW fish was dominated by *Vibrio* (10.92%), *Staphylococcu*s (10.60%), and *Acinetobacter* (9.25%).

### 3.3. Microbes from the environment could be found in fish gut microbiota

Two dominant genera *Plesiomonas* and *Cetobacterium* in FW fish guts were also found in the FW environment. *Acinetobacter* could also be found in FW fish guts and the environment. *Vibrio* was dominant in both SW fish guts and the environment. These results showed a correlation between environments and fish gut microbiota (Figure 3B).

### 3.4. Salinity-dependent abundance of the bacterial genus in fish gut

**FIGURE 4:**
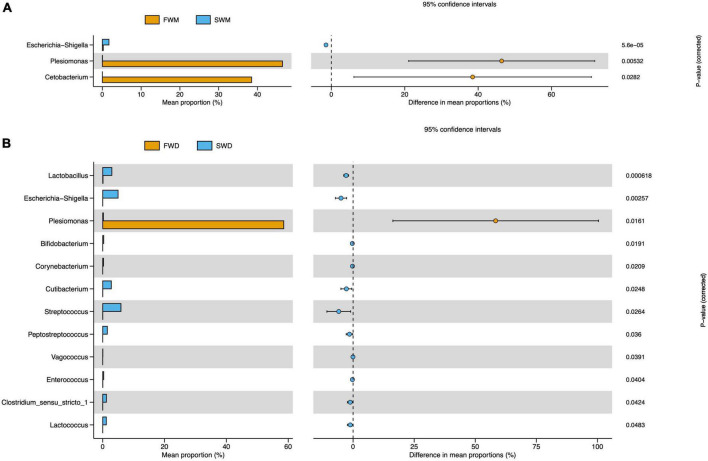
STAMP analysis revealed differentially abundant species between **(A)** FWM and SWM and **(B)** FWD and SWD. FWD, freshwater digesta samples; SWD, seawater digesta samples; FWM, freshwater mucosa samples; SWM, seawater mucosa samples.

Mucosa- and digesta-associated microbiota are affected by salinity. The most abundant bacteria in the mucosa of FW fish compared to SW fish were *Plesiomonas* and *Cetobacterium*, according to statistical analysis of metagenomic profiles (STAMP), whereas *Escherichia-Shigella* was the most prevalent in SW mucosa samples (Figure 4A). Salinity also changed the abundance of bacterial species in the digesta-associated microbiota between the FW and SW individuals. According to STAMP analysis, the abundance of *Plesiomonas* in the digesta of FW fish was significantly higher than that in the digesta of SW fish. On the other hand, *Lactobacillus, Escherichia-Shigella, Bifidobacterium, Corynebacterium, Cutibacterium, Streptococcus, Peptostreptococcus, Vagococcus, Enterococcus, Clostridium sensu stricto 1*, and *Lactococcus* were more abundant in the digesta of the SW fish than in that of FW individuals (Figure 4B).

### 3.5. Predictive functional analysis of microbiota

**FIGURE 5:**
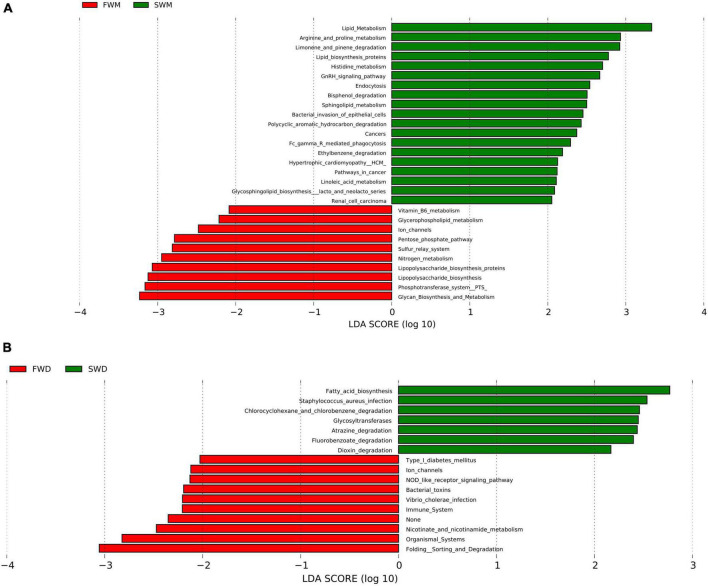
Comparisons of predictive KEGG pathways between **(A)** mucosa-associated and **(B)** digesta-associated microbiota of FW and SW individuals. FWM, freshwater mucosa samples; SWM, seawater mucosa samples; FWD, freshwater digesta samples; SWD, seawater digesta samples.

When predictive functional pathways were analyzed between the mucosa-associated microbiota of FW and SW individuals, 29 KEGG pathways were found to be differentially expressed. Metabolism, endocrine system, ion transporters, xenobiotic degradation, and glycan synthesis were all involved in these pathways. FW fish had significantly higher levels of ion channels, sulfur relay systems, vitamin B6 metabolism, and lipopolysaccharide biosynthesis, whereas SW fish had significantly higher levels of arginine, proline, and histidine metabolism, lipid biosynthesis proteins, and sphingolipid metabolism (Figure 5A).

In the digesta-associated microbiota from FW- and SW-acclimated fish, 17 pathways were found to be significantly different. These pathways included immune, metabolism, environmental adaptation, xenobiotic biodegradation, infectious disease, signal transduction, cell signaling, and growth and biosynthesis of secondary metabolites. NOD-like receptor signaling, *Vibrio cholerae* infection, and ion channel pathways were highly expressed in FW digesta microbiota, whereas fatty acid biosynthesis, atrazine degradation, and *Staphylococcus aureus* infection pathways were highly expressed in SW individuals (Figure 5B).

### 3.6. Correlation between microbiota and gene expression of cytokines

We analyzed the mRNA expression of five cytokines with pro- and anti-inflammatory functions in the same individuals and found that salinity had no effect on the expression level. Next, we analyzed the correlation between cytokine gene expression and the digesta- or mucosa-associated microbiota using Spearman’s correlation.

**FIGURE 6:**
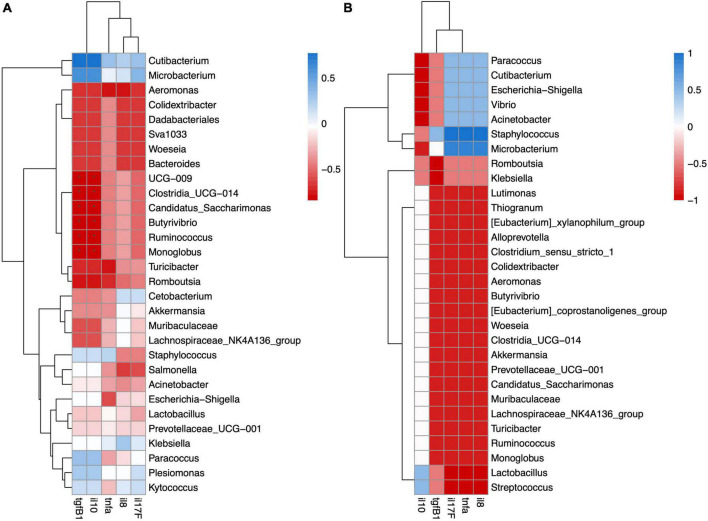
Spearman correlation analysis revealed significant positive or negative relation between cytokine gene expression and mucosa-associated microbiota in **(A)** FW and **(B)** SW individuals.

In the mucosa-associated microbiota of FW fish, UCG-009, Clostridia_UCG-014, *Candidatus Saccharimonas, Butyrivibrio, Ruminicoccus*, and *Monoglobus* were significantly negatively correlated with anti-inflammatory cytokines *tgfβ1* and *il10* (Figure 6A). Although the mucosa-associated microbiota of the SW fish did not show any significant correlation with cytokine gene expression, pathogens like genera *Vibrio* and *Escheria-shigella* had negative correlations with anti-inflammatory cytokines *tgfβ1* and *il10* and positive correlations with pro-inflammatory cytokines il8, *tnfα*, and il17F (Figure 6B).

**FIGURE 7:**
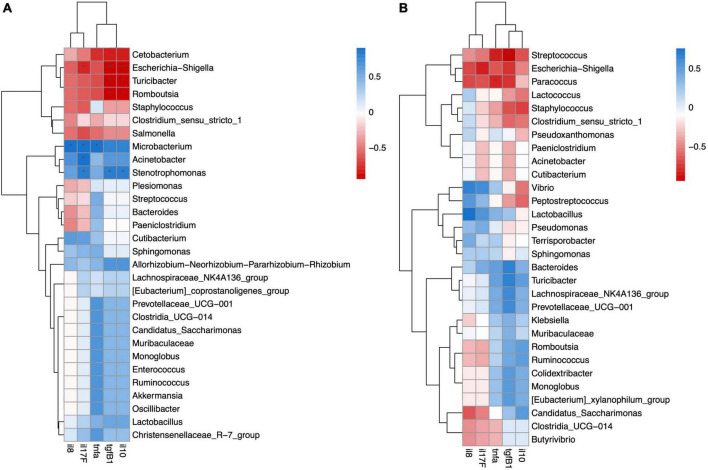
Spearman correlation analysis revealed significant positive or negative relation between cytokine gene expression and digesta-associated microbiota in **(A)** FW and **(B)** SW individuals.

In the digesta-associated microbiota of FW fish, *Escherichia-Shigella, Turicibacter*, and *Romboutsia* were significantly negatively correlated with the anti-inflammatory cytokines *tgfβ1* and *il10. Escherichia-Shigella* was also significantly negatively related to *il17F*. *Microbacterium* was significantly positively correlated with *il8, il17F*, and *tnfα. Acinetobacter* was significantly positively correlated with *il17F. Stenotrophomonas* was significantly positively correlated with *il17F*, *tgfβ1*, and *il10* (Figure 7A). In the digesta-associated microbiota of SW fish, *Streptococcus* was significantly negatively correlated with *tgfβ1* and *tnfα. Paracoccus* was significantly negatively correlated with *tnfα* expression (Figure 7B).

## 4. Discussion

As a catadromous fish, Asian sea bass migrates from SW to FW and matures (Jerry, 2013). A gill and kidney transcriptome study on FW and SW transfer in Asian sea bass revealed that major physiological pathways were affected by this transfer to acclimate according to the new environment (Vij et al., 2020). In contrast, when anadromous Atlantic salmon were transferred from FW to SW, a transcriptome study showed that there were significant alterations in transcripts related to the immune response (Johansson et al., 2016). In addition, SW transfer has been reported to change the skin and gut microbiota of Atlantic salmon (Lokesh and Kiron, 2016; Dehler et al., 2017). From these studies, it can be speculated that host-microbe interactions might occur during salinity transfer in anadromous fish. When euryhaline fish were sampled from different salinity groups, there was a clear change in the microbiota (Schmidt et al., 2015; Zhang et al., 2016). Taken together, these studies strongly support the idea that salinity affects fish microbiota, although the mechanisms underlying this change remain to be elucidated. However, this host-microbe interaction has not been elucidated in catadromous fish until now. Studies on mammals and other vertebrates have revealed that immune responses are correlated with microbiota homeostasis (Schirmer et al., 2016; Fuess et al., 2021). Mucosa- and digesta-associated microbiota play different roles in host physiology. Mucosa-associated microbiota is more important for host physiology because they colonize the intestine and maintain a symbiotic relationship (Wang et al., 2010; Yano et al., 2015). The intestine is an important organ in fish because it is in direct contact with the environment (Whittamore, 2012). Salinity has been found to change intestinal function, including water and ion transport (Whittamore, 2012). In this study, we focused on the effects of FW transfer on changes in microbiota and cytokine gene expression in the intestines of Asian sea bass. Based on previous studies, we hypothesized that FW transfer would change the gut microbiota of the Asian sea bass. Although the effect of salinity on fish gut microbiota has been reported previously, all of them have focused on digesta-associated microbiota, including studies on Asian sea bass (Dehler et al., 2017; Zheng et al., 2019). In this study, we simultaneously analyzed the mucosa- and digesta-associated microbiota to determine the effects of salinity on the composition of the gut microbiota. As cytokines are major drivers of microbiota regulation (Schirmer et al., 2016), we also analyzed cytokine gene expression in the same individuals to determine the correlation between microbiota composition and immune responses in the intestine. In this study, we found that FW transfer changed the gut microbiota of the Asian sea bass. We also found that salinity had differential effects on mucosa- and digesta-associated microbiota. In contrast, a previous study did not find any effect of salinity on the gut microbiota (digesta) of FW- and SW-acclimated Asian sea bass (Zheng et al., 2019) which is contrary to almost all studies on the effect of salinity on fish microbiota. Moreover, specific bacterial genera were positively or negatively correlated with cytokine gene expression.

Proteobacteria is the dominant phylum in the fish gut microbiota (Egerton et al., 2018). In a starvation study conducted on Asian sea bass, the FW-acclimated control group (mucosa and digesta mixed for analyses) was dominated by Proteobacteria, Firmicutes, Bacteroidetes, and Fusobacteria (Xia et al., 2014). In a study of SW-acclimated Asian sea bass, the authors found that Proteobacteria, Cyanobacteria, and Firmicutes were dominant in mucosa samples, and Proteobacteria, Fusobacteria, and Firmicutes were dominant in digesta samples (Apper et al., 2016). The dominant phyla found in different salinity groups in previous studies could also be found in the present study.

There was a significant difference in the alpha diversity of the digesta-associated microbiota between FW- and SW-acclimated fish in the current study, both in terms of microbial richness and diversity. Microbial richness was also high in SW-acclimated Atlantic salmon after being transferred from FW (Lokesh and Kiron, 2016), whereas the alpha diversity of the intestinal microbiota of Atlantic salmon was higher in FW-acclimated individuals (Dehler et al., 2017). This may be related to the opposite life cycle of Atlantic salmon (anadromous) and Asian sea bass (catadromous), although there is a gap in knowledge regarding how catadromous and anadromous fish microbiota change during their migration according to their life cycle. When Nile tilapia was cultured in environments with salinity higher than their preference, bacterial richness and diversity were found to decrease significantly (Zhang et al., 2016). This implies that although euryhaline fish can survive in a wide range of salinities, the effects of salinity on fish gut microbiota largely depend on their salinity preference, type of euryhalinity, and osmoregulatory capabilities. In the present study, beta diversity showed that individuals from the same groups clustered together based on their salinity or microbiota sample types. This implies that the fish microbiota in FW and SW differed. It also showed that mucosa- and digesta-associated microbiota were different based on beta diversity. Similar results were also found in the yellow drum (*Nibea albiflora*), as fish from different salinities were clustered into different groups (Tian et al., 2020).

ANOSIM analysis in this study confirmed that mucosa- and digesta-associated microbiota were significantly different between FW- and SW-acclimated Asian sea basses. In the FW group, the abundance of *Plesiomonas* and *Cetobacterium* was significantly higher than that in the SW group (in both the intestinal mucosa and digesta). Larsen et al. (2014) analyzed the gut microbiota of three species of FW fish collected from their natural habitats and found that *Plesiomonas* and *Cetobaterium* were the dominant genera (Larsen et al., 2014). *Cetobacterium* is a microaerotolerant, gram-negative, rod-shaped fermentative bacterium. It has also been detected in the gut microbiota of many other fish species (Tsuchiya et al., 2008; Silva et al., 2011; Di Maiuta et al., 2013). *Cetobacterium somerae* isolated from fish was found to be able to produce vitamin B_12_ (Tsuchiya et al., 2008). Depletion of *Cetobacterium* by antibiotic treatment caused pupfish to remain in paradoxical anaerobism for a longer period of time (Bhute et al., 2020). The authors suggested that *Cetobacterium* may play a role in the paradoxical anaerobism of pupfish. *Cetobacterium* seems to be an important commensal for FW fish physiology. The potential role of this bacterium in fish physiology is intriguing and is worth studying in the future. *Plesiomonas* is a pathogen for human (Brenden et al., 1988). *Plesiomonas shigelloides* was the first *Plesiomonas* species studied in fish and was found to be a pathogen for the FW inhabitant silver carp (Behera et al., 2018). More studies are required to unravel the interaction between *Plesiomonas* and fish to gain a deeper understanding of the roles and effects of this bacterium in fish intestines. Therefore, our finding of a higher abundance of *Plesiomonas* and *Cetobacterium* in FW Asian sea bass is in agreement with previous studies on the fish microbiota. These two bacteria might play important roles in the physiology of FW-acclimated Asian sea bass.

The abundance of pathogenic like bacteria *Escherichia-Shigella, Streptococcus, Bifidobacterium Vibrio* was higher in SW-acclimated sea bass gut microbiota. *Streptococcus* has been previously studied in fish as a pathogen of both marine and FW species (Eldar et al., 1995; Zlotkin et al., 1998; Evans et al., 2006). *Escherichia-Shigella* was found to be a part of the core microbiota of four carnivorous fish from the South China Sea (Gao et al., 2020). *Bifidobacterium* was also found to be the core microbiota in gilthead sea bream (*Sparus aurata*) (Piazzon et al., 2019). Both pathogenic and probiotic *Vibrio* spp. have been reported (Vandenberghe et al., 2003). *Vibrio* was also found to be the dominant genus in the SW-acclimated yellow drum (Tian et al., 2020). So, these bacteria could be common commensals of marine fish gut microbiota.

In the current study, the dominant gut microbiota (both in the mucosa and digesta) of Asian sea bass was also found in the water microbiota. The gut microbiota of tilapia larvae was found to change with rearing water microbiota (Giatsis et al., 2015). Minich et al. (2020) reported that the water microbiota affects the microbiota of the gut digesta and skin of Atlantic salmon. A recent study on several species of economically important fish further revealed that OTU in the fish gut microbiota could be found in their natural habitat water (Zeng et al., 2020). Changes in the water microbiota can alter the composition of the fish gut microbiota by microbe-microbe interactions in the gut (Coyte and Rakoff-Nahoum, 2019). Fish intestines may also have a selective approach to balancing the gut microbiota by changing their physiological mechanisms according to the ambient salinities of the environments they inhabit (Zhang et al., 2019). On the other hand, Schmidt et al. (2015) reported the opposite findings. They found that in Mexican mollies there was no correlation between water microbiota and fish microbiota, and fish dominant microbiota could not be found in the water microbiota. Although there are contradictory findings in the literature related to the effect of water microbiota on the fish microbiota, our results seem to support that the water microbiota and fish gut microbiota have significant interactions in both the mucosa and digesta. The underlying mechanisms of gut microbiota changes under different ambient salinities deserve more attention from ecological and aquaculture perspectives.

Microbial predictive functions were found to be differentially abundant in the different salinity groups in this study. In SW-acclimated fish microbiota amino acid and fatty acid synthesis was higher. Amino acids can function as osmolytes. Similar result was also found in Senegalese sole (*Solea senegalensis*), where proline was also higher in the blood plasma of high-salinity acclimated fish than in FW ones (Aragão et al., 2010). A transcriptome study of spotted sea bass (*Dicentrarchus punctatus*) revealed that lipid metabolism-related genes are highly expressed in SW-acclimated individuals (Zhang et al., 2017). Dietary vitamin B6 affected oxidative stress, growth, and gut microbiota of golden pompano (*Trachinotus ovatus*) (Huang et al., 2019). In our study, vitamin B6 was also higher in FW Asian sea bass. These changes in the microbiota functions might have happened due to the following three reasons: (i) a result of microbial adaptation strategy to different water salinity; (ii) to provide physiological benefits to the fish to adapt to different environments; (iii) both (i) and (ii). Microbial functions under changing environments in aquatic animals should be further studied.

Fish have both adaptive and innate immune systems (Zhu et al., 2013). Although salinity affects the fish immune system, this issue has not been explored that much (Bowden, 2008). In this study, we found that specific bacterial genera were positively or negatively correlated with the expression of cytokine genes. Host gene expression and microbiota have bi-directional interaction to maintain the homeostasis (Nichols and Davenport, 2021). Large-scale gut microbiota and head kidney transcriptome analyses of sticklebacks (*Gasterosteus aculeatus*) revealed a significant correlation between host immune gene expression and microbiota composition (Fuess et al., 2021).

In the mucosa-associated microbiota of the FW group, UCG-009, *Clostridia_UCG-014, Candidatus Saccharimonas, Butyrivibrio, Ruminococcus*, and *Monoglobus* were significantly negatively related with *il10* and *tgfβ1*. *Candidatus Saccharimonas* is associated with human diseases such as IBD and gingivitis etc. (dos Santos Cruz et al., 2020). *Monoglobus* was positively correlated with serum triglyceride and liver MDA content in mice (Zhang et al., 2020). Therefore, this bacterium might cause oxidative stress in the host by suppressing the expression of *il10* and *tgfβ1. Ruminococcus* was also found in Asian sea bass in high abundance after feeding with poultry by-products (Chaklader et al., 2021). In contrast, in the mucosa-associated microbiota of the SW group, there was no significant correlation between the microbes and gene expression of cytokines. Analyses of the correlation between microbiota and host gene expression in sticklebacks also revealed the immune-suppressive effects of particular microbial families (Fuess et al., 2021).

In the digesta-associated microbiota of the FW group, *Escherichia-Shigella* was significantly negatively related to *il10, tgfβ1*, and *il17F. Escherichia-shigella* is considered as a pathogen to humans and animals (Falkow et al., 1963; Zhao et al., 2022). Its pathogenic function may explain its negative interaction with anti-inflammatory cytokines. *Turicibacter* and *Romboutsia* were significantly negatively correlated with *il10* and *tgfβ1* expression. *Romboutsia* is commonly found in fish and humans (Ricaboni et al., 2016; Abdelhamed et al., 2019), although the role of *Romboutsia* in the fish gut has not yet been studied. *Microbacterium* was significantly positively correlated with *il8, il17F*, and *tnfα. Microbacteria* have been isolated from human clinical samples and meat products (Gardner, 1966; Gneiding et al., 2008). This bacterium has also been found to be a causative agent of bacteremia in humans (Lau et al., 2002). As this bacterium has a pathogenic nature in humans, this may explain its positive correlation with pro-inflammatory cytokines. *Acinetobacter* was significantly positively correlated with *the il17F* levels. Some species of *Acinetobacter have* already been identified as pathogens of fish, which may be a reason for its positive correlation with pro-inflammatory *il17F* (Kozińska et al., 2014; Malick et al., 2020). *Stenotrophomonas* was significantly positively correlated with *il17F*, *tgfβ1*, and *il10*. *Stenotrophomonas* has been identified as an opportunistic pathogen in channel catfish (Geng et al., 2010). In the digesta-associated microbiota of SW fish, *Streptococcus* was negatively related to *tgfβ1* and *tnfα*, which may be due to the pathogenic nature of this bacterium (Eldar et al., 1995). *Paracoccus* was significantly negatively correlated with *tnfα* expression. *Paracoccus* has been found to induce skin coloration in red sea bream (Kurnia et al., 2010). It has also been used as a probiotic in sea cucumbers and can enhance intestinal innate immunity through the NF-κB signaling pathway (Yang et al., 2015). In sticklebacks, it was also found that some pathogenic bacteria were negatively related to immune gene expression, as the pathogen effect on immune response may vary under different conditions (Fuess et al., 2021).

## 5. Conclusion

This study revealed that FW transfer affected both mucosa- and digesta-associated microbiota of the Asian sea bass. Salinity had differential effects on mucosa- and digesta-associated microbiota. In addition, dominant fish microbiota was also found in the water, suggesting a microbiota continuum between water and fish. Several bacterial genera were positively or negatively correlated with cytokine gene expression. Future studies will examine the effects of these bacteria on the physiological mechanisms of Asian sea bass, including growth, immunity, and metabolism.

## Data availability statement

The datasets presented in this study can be found in online repositories. The names of the repository/repositories and accession number(s) can be found below: https://www.ncbi.nlm.nih.gov/, PRJNA899487.

## Ethics statement

The experimental protocol was reviewed and approved by the Institutional Animal Care and Use Committee (IACUC No. 108-137) of the National Chung Hsing University, Taichung, Taiwan.

## Author contributions

SM, Y-YC, Y-PC, and T-HL designed the experiments. SM performed the experiments. SM and C-HL analyzed the data. SM and T-HL wrote the manuscript. All authors read and approved the final manuscript.

## Footnotes

1
https://primer3plus.com/cgi-bin/dev/primer3plus.cgi

2
https://www.ncbi.nlm.nih.gov/tools/primer-blast/
