# Supplementary material for: Freshwater transfer affected intestinal microbiota with correlation to cytokine gene expression in Asian sea bass
Source: Front Microbiol. 2023 Apr 6;14:1097954. doi: 10.3389/fmicb.2023.1097954 (PMC10117908; doi:10.3389/fmicb.2023.1097954)
Supplement: Supplementary file 1 [file Data_Sheet_1.docx]

Table S1. Primers used for the quantitative real-time PCR (qPCR).

Gene Primer sequence (5´ to 3´) Amplicon size (bp) Ta (°C) Efficiency (%) Accession number

*elf1α* F: GTTGCCTTTGTCCCCATCTC 130 65 99 GU188685.1

R: CTTCCAGCAGTGTGGTTCCA

*tgfβ1* F: AGCCACTGCCCAATCCTTTAC 130 65 96 XM_018665504.1

R: TATGTTTGAAGGGAGGCAGAGC

*tnfα* F: GGCCGGAAATGATGATGCAAT 109 65 102 XM_018699809

R: GCTTTGCTGCTGATTCGCTT

*il10* F: TCTGCATCCAGAAGATCTCAGC 119 65 105 XM_018686737.1

R: TGTTGCACATTGGACTGCAC

*il7F* F: GACACCTATGTGGCATCTCG 189 65 95 XM_018694770.1

R: CCTTGCTTTTCTCCCCCTTTTG

*il8* F: GCATCATCAAGGAGAGAAAGCC 199 65 96 XM_018695863.1

R: AAGGTTCTTCAAGGTGTCTGCT

F, forward primer; R, reverse primer; Ta, annealing temperature. The accession number of the target sequence were obtained from the National Center for Biotechnology Information (<https://www.ncbi.nlm.nih.gov/>)

Table S2. Relative abundance (%) of top 30 phylum

Table S3. Relative abundance (%) of top 38 genus


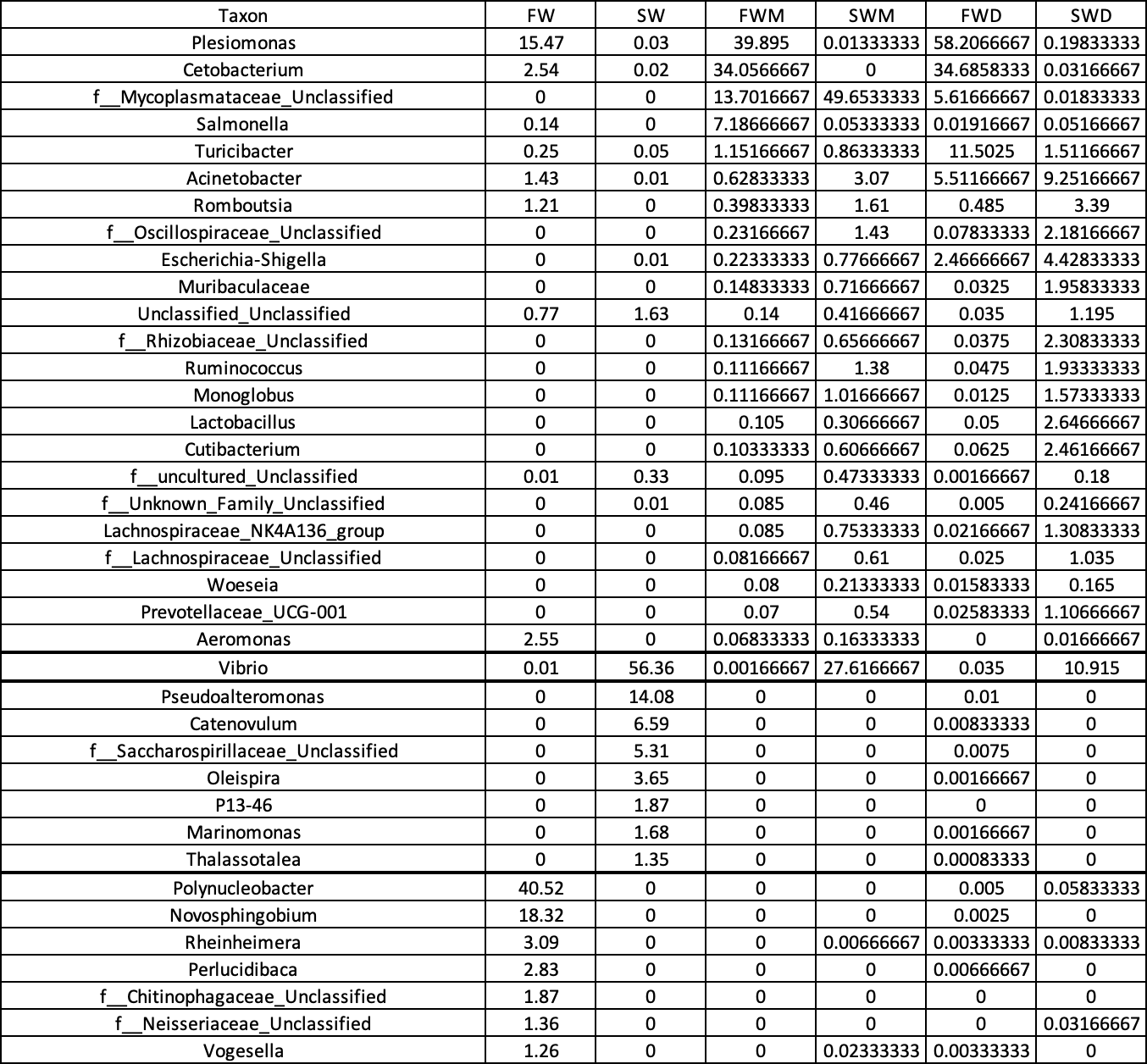

Figure S1. Rarefaction curve plotted against observed OTUs.

Figure S2: Relative mRNA expression of cytokines between FW- and SW acclimated Asian sea bass intestines. Mann- Whitney U-test was used for statistical analysis. Salinity did not have any significant effects on cytokine gene expressions. n=6
